# Aqua­[tris­(2-{5-[(4-methyl­phenyl)diazen­yl]-2-oxidobenzyl­idene­amino}­eth­yl)amine]­samarium(III) acetonitrile monosolvate

**DOI:** 10.1107/S1600536811054961

**Published:** 2012-01-07

**Authors:** Sadegh Salehzadeh, Mahsa Mahdavian, Mehdi Khalaj

**Affiliations:** a Faculty of Chemistry, Bu-Ali Sina University, Hamedan, Iran; b Chemistry Department, Isalmic Azad University, Buinzahra Branch, Qazvin, Iran

## Abstract

In the title compound, [Sm(C_48_H_45_N_10_O_3_)(H_2_O)]·CH_3_CN, the Sm^III^ ion is coordinated by the hepta­dentate tris­(2-{5-[(4-methyl­pheny)diazen­yl]-2-oxidobenzyl­idene­amino}­eth­yl)amine trianionic ligand and a water mol­ecule. The resulting SmN_4_O_4_ coordination polyhedron is a distorted square anti­prism. In the crystal, complex mol­ecules are linked by O—H⋯O hydrogen bonds.

## Related literature

For related samarium complexes, see: Salehzadeh *et al.* (2005 ▶); Kanesato *et al.* (2004 ▶). For azo compounds, see: Khandar & Nejati (2000 ▶). For the synthesis of the ligand, see: Salehzadeh *et al.* (2011 ▶). 
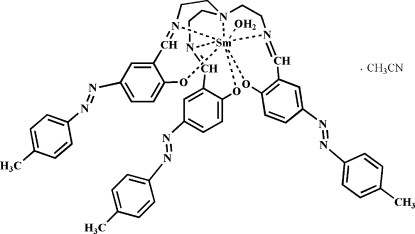


## Experimental

### 

#### Crystal data

[Sm(C_48_H_45_N_10_O_3_)(H_2_O)]·C_2_H_3_N
*M* *_r_* = 1019.36
Monoclinic, 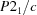

*a* = 19.9785 (5) Å
*b* = 20.4849 (4) Å
*c* = 11.4683 (8) Å
β = 95.3750 (14)°
*V* = 4672.9 (4) Å^3^
*Z* = 4
Mo *K*α radiation
μ = 1.31 mm^−1^
*T* = 150 K
0.35 × 0.11 × 0.08 mm

#### Data collection

Nonius KappaCCD diffractometer
Absorption correction: multi-scan (*SORTAV*; Blessing, 1995 ▶) *T*_min_ = 0.727, *T*_max_ = 0.907
32101 measured reflections
10613 independent reflections
5694 reflections with *I* > 2σ(*I*)
*R*_int_ = 0.105

#### Refinement

*R*[*F* ^2^ > 2σ(*F* ^2^)] = 0.056
*wR*(*F* ^2^) = 0.128
*S* = 1.02
10613 reflections
598 parameters
2 restraints
H-atom parameters constrained
Δρ_max_ = 2.91 e Å^−3^
Δρ_min_ = −0.96 e Å^−3^

### 

Data collection: *COLLECT* (Nonius, 2002 ▶); cell refinement: *DENZO-SMN* (Otwinowski & Minor, 1997 ▶); data reduction: *DENZO-SMN*; program(s) used to solve structure: *SIR92* (Altomare *et al.*, 1994 ▶); program(s) used to refine structure: *SHELXTL* (Sheldrick, 2008 ▶); molecular graphics: *PLATON* (Spek, 2009 ▶); software used to prepare material for publication: *SHELXTL*.

## Supplementary Material

Crystal structure: contains datablock(s) I, global. DOI: 10.1107/S1600536811054961/hb6567sup1.cif

Structure factors: contains datablock(s) I. DOI: 10.1107/S1600536811054961/hb6567Isup2.hkl

Additional supplementary materials:  crystallographic information; 3D view; checkCIF report

## Figures and Tables

**Table 1:** Selected bond lengths (Å)

Sm1—O2	2.302 (4)
Sm1—O3	2.321 (4)
Sm1—O1	2.346 (3)
Sm1—O4	2.432 (3)
Sm1—N2	2.556 (4)
Sm1—N3	2.605 (5)
Sm1—N4	2.671 (5)
Sm1—N1	2.753 (5)

**Table 2:** Hydrogen-bond geometry (Å, °)

*D*—H⋯*A*	*D*—H	H⋯*A*	*D*⋯*A*	*D*—H⋯*A*
O4—H4*OA*⋯O1^i^	0.93	1.76	2.624 (5)	153
O4—H4*OB*⋯O3^i^	0.83	2.00	2.761 (5)	153

Symmetry code: (i) 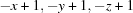.

**Fig. 1.**
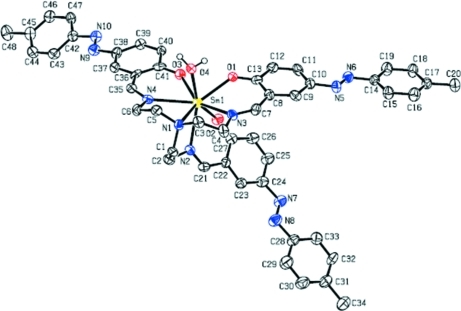
A view of the structure of the title complex, with displacement ellipsoids drawn at the 50% probability level.

## supplementary crystallographic information

### Comment

The wide spread application of azo compounds and their metal complexes have attracted the interest of many investigators. In this work the synthesis and X-ray crystal structure of the samarium complex containing azo Schiff base ligand was reported. In the resulting complex the central Sm atom is eight-coordinate. The average of Sm–Nimine bond lengths [2.61 (5)Å] is shorter than Sm–Ntert bond length [2.75 (5)Å], also the average of Sm–Ophenolic bond length [2.32 (4)Å] is shorter than Sm–OH_2_O bond length [2.43 (3)Å]. The angles N2–Sm–N4, N2–Sm–N3 and N3–Sm–N4 have values of 85.17 (14), 83.96 (14) and 130.23 (15)°, respectively.

### Experimental

Azo Schiff base ligand H_3_*L*^1^(tris({[5-(4-methylphenylazo)salicylidene]amino}ethyl)amine) was synthesized according to the literature procedure (Salehzadeh *et al.*, 2011). Then to a solution of ligand H_3_*L*^1^ (1 mmol) in methanol:choloform (1:1.5) (40 ml) was added Sm(NO_3_)_3_.6H_2_O, (1 mmol) in methanol (10 ml). Then triethylamine (3 mmol) was added to the mixture and was refluxed for 24 h. The resulting precipitate was filtered, washed with diethyl ether, and dried in vacuum. Orange needles were obtained by slow evaporationfrom an acetonitrile solution at room temperature after 10 h.

### Refinement

All H atoms bonded to C atoms were positioned geometrically [C–H = 0.95 to 0.99 Å] and were refined using a riding-model approximation, with *U*_iso_(H) = 1.2 *U*_eq_ (C) or 1.5 *U*_eq_ (C_methyl_) The H atoms bonded to O atoms were located in a difference Fourier map and then included in their 'as found' positions and refined as riding with *U*_iso_(H) = 1.5 *U*_eq_ (O).

### Crystal data

**Table:**

[Sm(C_48_H_45_N_10_O_3_)(H_2_O)]·C_2_H_3_N	*F*(000) = 2084
*M**_r_* = 1019.36	*D*_x_ = 1.449 Mg m^−^^3^
Monoclinic, *P*2_1_/*c*	Mo *K*α radiation, λ = 0.71073 Å
Hall symbol: -P 2ybc	Cell parameters from 32101 reflections
*a* = 19.9785 (5) Å	θ = 2.7–27.5°
*b* = 20.4849 (4) Å	µ = 1.31 mm^−^^1^
*c* = 11.4683 (8) Å	*T* = 150 K
β = 95.3750 (14)°	Needle, orange
*V* = 4672.9 (4) Å^3^	0.35 × 0.11 × 0.08 mm
*Z* = 4	

### Data collection

**Table:**

Nonius KappaCCD diffractometer	10613 independent reflections
Radiation source: fine-focus sealed tube	5694 reflections with *I* > 2σ(*I*)
graphite	*R*_int_ = 0.105
Detector resolution: 9 pixels mm^-1^	θ_max_ = 27.5°, θ_min_ = 2.7°
φ scans and ω scans with κ offsets	*h* = −25→25
Absorption correction: multi-scan (*SORTAV*; Blessing, 1995)	*k* = −26→21
*T*_min_ = 0.727, *T*_max_ = 0.907	*l* = −14→14
32101 measured reflections	

### Refinement

**Table:**

Refinement on *F*^2^	Primary atom site location: structure-invariant direct methods
Least-squares matrix: full	Secondary atom site location: difference Fourier map
*R*[*F*^2^ > 2σ(*F*^2^)] = 0.056	Hydrogen site location: inferred from neighbouring sites
*wR*(*F*^2^) = 0.128	H-atom parameters constrained
*S* = 1.02	*w* = 1/[σ^2^(*F*_o_^2^) + (0.0418*P*)^2^ + 3.712*P*] where *P* = (*F*_o_^2^ + 2*F*_c_^2^)/3
10613 reflections	(Δ/σ)_max_ = 0.002
598 parameters	Δρ_max_ = 2.91 e Å^−^^3^
2 restraints	Δρ_min_ = −0.96 e Å^−^^3^

### Special details

**Table:**

Geometry. All e.s.d.'s (except the e.s.d. in the dihedral angle between two l.s. planes) are estimated using the full covariance matrix. The cell e.s.d.'s are taken into account individually in the estimation of e.s.d.'s in distances, angles and torsion angles; correlations between e.s.d.'s in cell parameters are only used when they are defined by crystal symmetry. An approximate (isotropic) treatment of cell e.s.d.'s is used for estimating e.s.d.'s involving l.s. planes.
Refinement. Refinement of *F*^2^ against ALL reflections. The weighted *R*-factor *wR* and goodness of fit *S* are based on *F*^2^, conventional *R*-factors *R* are based on *F*, with *F* set to zero for negative *F*^2^. The threshold expression of *F*^2^ > σ(*F*^2^) is used only for calculating *R*-factors(gt) *etc*. and is not relevant to the choice of reflections for refinement. *R*-factors based on *F*^2^ are statistically about twice as large as those based on *F*, and *R*- factors based on ALL data will be even larger.

### Fractional atomic coordinates and isotropic or equivalent isotropic displacement parameters (Å^2^)

**Table:**

	*x*	*y*	*z*	*U*_iso_*/*U*_eq_	
Sm1	0.536045 (15)	0.623474 (13)	0.44296 (3)	0.02530 (10)	
O1	0.60176 (19)	0.53292 (16)	0.5056 (3)	0.0277 (9)	
O2	0.6177 (2)	0.66748 (17)	0.5748 (3)	0.0327 (10)	
O3	0.48049 (19)	0.59716 (17)	0.6055 (3)	0.0303 (9)	
O4	0.47508 (18)	0.52949 (16)	0.3581 (3)	0.0277 (9)	
H4OA	0.4386	0.5177	0.3985	0.042*	
H4OB	0.4993	0.4967	0.3599	0.042*	
N1	0.4901 (2)	0.6597 (2)	0.2184 (4)	0.0281 (11)	
N2	0.5619 (2)	0.7406 (2)	0.3845 (4)	0.0289 (11)	
N3	0.6185 (2)	0.5925 (2)	0.2888 (4)	0.0302 (12)	
N4	0.4074 (2)	0.6606 (2)	0.4136 (4)	0.0279 (11)	
N5	0.8661 (2)	0.4506 (2)	0.4962 (5)	0.0354 (13)	
N6	0.8946 (2)	0.4311 (2)	0.5927 (4)	0.0342 (12)	
N7	0.8549 (3)	0.8034 (2)	0.5355 (5)	0.0382 (13)	
N8	0.8538 (3)	0.8422 (3)	0.4506 (5)	0.0455 (14)	
N9	0.2698 (3)	0.6663 (2)	0.8586 (5)	0.0358 (13)	
N10	0.2712 (2)	0.6490 (2)	0.9652 (5)	0.0345 (12)	
C1	0.5013 (3)	0.7298 (3)	0.1921 (5)	0.0338 (15)	
H1A	0.4619	0.7466	0.1422	0.041*	
H1B	0.5411	0.7337	0.1474	0.041*	
C2	0.5120 (3)	0.7715 (3)	0.3021 (5)	0.0377 (16)	
H2A	0.5276	0.8155	0.2817	0.045*	
H2B	0.4691	0.7761	0.3380	0.045*	
C3	0.5274 (3)	0.6194 (3)	0.1397 (5)	0.0328 (14)	
H3A	0.5177	0.6352	0.0583	0.039*	
H3B	0.5117	0.5736	0.1427	0.039*	
C4	0.6018 (3)	0.6214 (3)	0.1721 (5)	0.0387 (15)	
H4A	0.6250	0.5969	0.1133	0.046*	
H4B	0.6176	0.6672	0.1722	0.046*	
C5	0.4181 (3)	0.6437 (3)	0.2033 (5)	0.0326 (15)	
H5A	0.4125	0.5959	0.2108	0.039*	
H5B	0.3996	0.6566	0.1236	0.039*	
C6	0.3791 (3)	0.6777 (3)	0.2921 (5)	0.0342 (15)	
H6A	0.3814	0.7256	0.2808	0.041*	
H6B	0.3313	0.6644	0.2805	0.041*	
C7	0.6715 (3)	0.5564 (3)	0.3002 (5)	0.0328 (14)	
H7A	0.6945	0.5508	0.2320	0.039*	
C8	0.7001 (3)	0.5235 (3)	0.4044 (5)	0.0313 (14)	
C9	0.7654 (3)	0.4989 (3)	0.4052 (5)	0.0331 (15)	
H9A	0.7876	0.5015	0.3356	0.040*	
C10	0.7990 (3)	0.4709 (3)	0.5041 (6)	0.0337 (15)	
C11	0.7647 (3)	0.4655 (3)	0.6047 (5)	0.0359 (15)	
H11A	0.7869	0.4467	0.6735	0.043*	
C12	0.7002 (3)	0.4866 (3)	0.6057 (5)	0.0360 (15)	
H12A	0.6783	0.4819	0.6752	0.043*	
C13	0.6645 (3)	0.5159 (3)	0.5048 (5)	0.0301 (14)	
C14	0.9629 (3)	0.4108 (3)	0.5910 (5)	0.0310 (15)	
C15	1.0002 (3)	0.4126 (3)	0.4959 (6)	0.0389 (16)	
H15A	0.9802	0.4276	0.4223	0.047*	
C16	1.0671 (3)	0.3925 (3)	0.5074 (6)	0.0457 (17)	
H16A	1.0924	0.3942	0.4415	0.055*	
C17	1.0973 (3)	0.3700 (3)	0.6141 (6)	0.0361 (15)	
C18	1.0590 (3)	0.3679 (3)	0.7083 (6)	0.0412 (16)	
H18A	1.0786	0.3524	0.7818	0.049*	
C19	0.9926 (3)	0.3881 (3)	0.6976 (6)	0.0436 (17)	
H19A	0.9672	0.3864	0.7635	0.052*	
C20	1.1696 (3)	0.3481 (3)	0.6284 (6)	0.0492 (19)	
H20A	1.1929	0.3646	0.5630	0.074*	
H20B	1.1714	0.3003	0.6294	0.074*	
H20C	1.1915	0.3652	0.7023	0.074*	
C21	0.6170 (3)	0.7718 (3)	0.4055 (5)	0.0316 (14)	
H21A	0.6210	0.8117	0.3646	0.038*	
C22	0.6739 (3)	0.7526 (3)	0.4846 (5)	0.0296 (14)	
C23	0.7329 (3)	0.7882 (3)	0.4776 (5)	0.0330 (15)	
H23A	0.7331	0.8229	0.4227	0.040*	
C24	0.7910 (3)	0.7745 (3)	0.5477 (5)	0.0360 (16)	
C25	0.7889 (3)	0.7256 (3)	0.6328 (6)	0.0425 (17)	
H25A	0.8280	0.7163	0.6837	0.051*	
C26	0.7309 (3)	0.6910 (3)	0.6435 (5)	0.0369 (16)	
H26A	0.7306	0.6590	0.7033	0.044*	
C27	0.6719 (3)	0.7014 (3)	0.5689 (5)	0.0315 (14)	
C28	0.9173 (3)	0.8707 (3)	0.4305 (5)	0.0380 (15)	
C29	0.9156 (4)	0.9195 (3)	0.3476 (6)	0.0535 (19)	
H29A	0.8739	0.9325	0.3079	0.064*	
C30	0.9742 (4)	0.9497 (3)	0.3219 (6)	0.055 (2)	
H30A	0.9723	0.9828	0.2637	0.066*	
C31	1.0354 (3)	0.9327 (3)	0.3795 (6)	0.0427 (17)	
C32	1.0367 (3)	0.8825 (3)	0.4607 (6)	0.0422 (16)	
H32A	1.0786	0.8690	0.4993	0.051*	
C33	0.9785 (3)	0.8517 (3)	0.4868 (6)	0.0407 (16)	
H33A	0.9804	0.8177	0.5433	0.049*	
C34	1.1002 (3)	0.9653 (3)	0.3524 (7)	0.061 (2)	
H34A	1.0977	0.9762	0.2689	0.091*	
H34B	1.1380	0.9355	0.3720	0.091*	
H34C	1.1069	1.0053	0.3989	0.091*	
C35	0.3633 (3)	0.6618 (2)	0.4880 (5)	0.0268 (14)	
H35A	0.3197	0.6765	0.4595	0.032*	
C36	0.3723 (3)	0.6432 (3)	0.6117 (5)	0.0291 (14)	
C37	0.3202 (3)	0.6589 (3)	0.6804 (5)	0.0313 (14)	
H37A	0.2827	0.6830	0.6461	0.038*	
C38	0.3215 (3)	0.6406 (3)	0.7962 (5)	0.0330 (15)	
C39	0.3762 (3)	0.6022 (3)	0.8448 (5)	0.0312 (14)	
H39A	0.3770	0.5873	0.9234	0.037*	
C40	0.4274 (3)	0.5865 (2)	0.7797 (5)	0.0265 (13)	
H40A	0.4635	0.5601	0.8126	0.032*	
C41	0.4271 (3)	0.6092 (2)	0.6628 (5)	0.0302 (14)	
C42	0.2206 (3)	0.6816 (3)	1.0250 (5)	0.0303 (14)	
C43	0.1884 (3)	0.7385 (3)	0.9839 (5)	0.0336 (15)	
H43A	0.1986	0.7576	0.9121	0.040*	
C44	0.1414 (3)	0.7670 (3)	1.0491 (6)	0.0382 (16)	
H44A	0.1187	0.8052	1.0201	0.046*	
C45	0.1265 (3)	0.7413 (3)	1.1555 (5)	0.0373 (16)	
C46	0.1601 (3)	0.6850 (3)	1.1949 (5)	0.0343 (15)	
H46A	0.1503	0.6660	1.2668	0.041*	
C47	0.2075 (3)	0.6564 (3)	1.1316 (5)	0.0313 (14)	
H47A	0.2313	0.6190	1.1618	0.038*	
C48	0.0761 (3)	0.7749 (3)	1.2262 (6)	0.0497 (18)	
H48A	0.0618	0.7447	1.2853	0.075*	
H48B	0.0368	0.7883	1.1739	0.075*	
H48C	0.0969	0.8135	1.2650	0.075*	
N1S	0.6088 (5)	0.5337 (4)	0.9036 (8)	0.100 (3)	
C1S	0.6652 (6)	0.5289 (4)	0.9230 (9)	0.076 (3)	
C2S	0.7374 (5)	0.5226 (4)	0.9526 (9)	0.088 (3)	
H2S1	0.7523	0.5551	1.0122	0.132*	
H2S2	0.7608	0.5298	0.8824	0.132*	
H2S3	0.7476	0.4787	0.9832	0.132*	

### Atomic displacement parameters (Å^2^)

**Table:**

	*U*^11^	*U*^22^	*U*^33^	*U*^12^	*U*^13^	*U*^23^
Sm1	0.02771 (17)	0.02548 (16)	0.02267 (17)	−0.00037 (14)	0.00209 (12)	0.00194 (15)
O1	0.026 (2)	0.026 (2)	0.032 (3)	0.0015 (16)	0.0031 (19)	0.0061 (17)
O2	0.034 (3)	0.037 (2)	0.027 (2)	−0.0067 (19)	−0.0024 (19)	−0.0014 (18)
O3	0.032 (2)	0.033 (2)	0.026 (2)	0.0010 (17)	0.0027 (18)	0.0015 (17)
O4	0.030 (2)	0.026 (2)	0.027 (2)	0.0019 (16)	0.0020 (18)	0.0005 (17)
N1	0.027 (3)	0.033 (3)	0.025 (3)	0.004 (2)	0.003 (2)	0.004 (2)
N2	0.030 (3)	0.028 (3)	0.028 (3)	0.002 (2)	0.001 (2)	0.002 (2)
N3	0.033 (3)	0.031 (3)	0.025 (3)	0.005 (2)	0.000 (2)	0.005 (2)
N4	0.033 (3)	0.024 (3)	0.026 (3)	−0.002 (2)	0.001 (2)	0.003 (2)
N5	0.028 (3)	0.041 (3)	0.036 (3)	0.006 (2)	0.000 (3)	0.001 (2)
N6	0.030 (3)	0.038 (3)	0.034 (3)	−0.001 (2)	−0.001 (3)	0.001 (2)
N7	0.036 (3)	0.048 (3)	0.029 (3)	−0.010 (2)	−0.005 (3)	0.001 (3)
N8	0.041 (4)	0.059 (4)	0.037 (4)	−0.009 (3)	0.002 (3)	0.011 (3)
N9	0.041 (3)	0.039 (3)	0.028 (3)	0.000 (2)	0.007 (3)	0.001 (2)
N10	0.035 (3)	0.038 (3)	0.031 (3)	0.001 (2)	0.003 (3)	−0.002 (2)
C1	0.030 (4)	0.032 (3)	0.038 (4)	0.002 (3)	−0.005 (3)	0.017 (3)
C2	0.039 (4)	0.031 (3)	0.042 (4)	0.000 (3)	−0.002 (3)	0.006 (3)
C3	0.040 (4)	0.041 (3)	0.017 (3)	0.007 (3)	0.001 (3)	0.007 (3)
C4	0.041 (4)	0.054 (4)	0.022 (3)	0.017 (3)	0.008 (3)	0.012 (3)
C5	0.035 (4)	0.037 (3)	0.025 (4)	0.000 (3)	0.000 (3)	0.007 (3)
C6	0.024 (3)	0.045 (4)	0.033 (4)	0.004 (3)	−0.002 (3)	0.011 (3)
C7	0.030 (4)	0.043 (4)	0.027 (4)	0.002 (3)	0.008 (3)	0.000 (3)
C8	0.029 (4)	0.032 (3)	0.032 (4)	0.003 (3)	−0.001 (3)	0.000 (3)
C9	0.045 (4)	0.031 (3)	0.023 (4)	0.006 (3)	0.008 (3)	0.004 (3)
C10	0.030 (4)	0.036 (3)	0.036 (4)	0.005 (3)	0.005 (3)	0.006 (3)
C11	0.036 (4)	0.039 (4)	0.031 (4)	0.009 (3)	−0.006 (3)	0.009 (3)
C12	0.036 (4)	0.043 (4)	0.030 (4)	−0.002 (3)	0.004 (3)	0.011 (3)
C13	0.033 (4)	0.028 (3)	0.029 (4)	−0.007 (3)	0.000 (3)	0.003 (3)
C14	0.029 (4)	0.030 (3)	0.035 (4)	0.001 (3)	0.006 (3)	−0.004 (3)
C15	0.036 (4)	0.049 (4)	0.031 (4)	0.007 (3)	−0.002 (3)	0.003 (3)
C16	0.042 (4)	0.056 (4)	0.039 (4)	0.004 (3)	0.007 (3)	−0.002 (3)
C17	0.027 (3)	0.031 (3)	0.049 (4)	−0.001 (3)	−0.002 (3)	−0.006 (3)
C18	0.031 (4)	0.055 (4)	0.036 (4)	0.007 (3)	−0.004 (3)	0.003 (3)
C19	0.037 (4)	0.059 (4)	0.035 (4)	0.007 (3)	0.003 (3)	0.010 (3)
C20	0.035 (4)	0.049 (4)	0.063 (5)	0.008 (3)	0.002 (4)	0.002 (4)
C21	0.038 (4)	0.032 (3)	0.025 (4)	−0.004 (3)	0.003 (3)	0.004 (3)
C22	0.024 (3)	0.030 (3)	0.035 (4)	0.000 (2)	0.002 (3)	−0.004 (3)
C23	0.035 (4)	0.032 (3)	0.033 (4)	−0.003 (3)	0.005 (3)	−0.003 (3)
C24	0.040 (4)	0.036 (4)	0.031 (4)	−0.009 (3)	−0.005 (3)	−0.002 (3)
C25	0.042 (4)	0.047 (4)	0.037 (4)	−0.009 (3)	−0.003 (3)	0.001 (3)
C26	0.044 (4)	0.043 (4)	0.023 (4)	−0.008 (3)	−0.002 (3)	0.004 (3)
C27	0.036 (4)	0.033 (3)	0.025 (4)	−0.004 (3)	0.004 (3)	−0.008 (3)
C28	0.035 (4)	0.047 (4)	0.032 (4)	−0.009 (3)	0.000 (3)	0.002 (3)
C29	0.043 (5)	0.068 (5)	0.048 (5)	−0.005 (4)	−0.003 (4)	0.016 (4)
C30	0.049 (5)	0.062 (5)	0.054 (5)	−0.010 (4)	0.002 (4)	0.018 (4)
C31	0.041 (4)	0.050 (4)	0.038 (4)	−0.009 (3)	0.012 (3)	−0.007 (3)
C32	0.025 (3)	0.060 (4)	0.043 (4)	−0.008 (3)	0.005 (3)	0.002 (4)
C33	0.042 (4)	0.049 (4)	0.031 (4)	0.004 (3)	0.003 (3)	0.006 (3)
C34	0.044 (5)	0.069 (5)	0.071 (6)	−0.008 (4)	0.013 (4)	0.006 (4)
C35	0.028 (4)	0.024 (3)	0.027 (4)	−0.003 (2)	−0.003 (3)	−0.004 (3)
C36	0.027 (3)	0.032 (3)	0.030 (4)	−0.015 (2)	0.012 (3)	−0.011 (3)
C37	0.027 (4)	0.031 (3)	0.036 (4)	−0.001 (3)	0.000 (3)	0.000 (3)
C38	0.030 (4)	0.043 (4)	0.027 (4)	0.001 (3)	0.008 (3)	−0.001 (3)
C39	0.033 (4)	0.033 (3)	0.027 (4)	−0.001 (3)	0.004 (3)	−0.002 (3)
C40	0.025 (3)	0.027 (3)	0.028 (4)	0.002 (2)	0.005 (3)	0.003 (3)
C41	0.045 (4)	0.026 (3)	0.019 (3)	−0.017 (3)	0.001 (3)	−0.003 (2)
C42	0.029 (4)	0.032 (3)	0.030 (4)	−0.001 (3)	−0.001 (3)	−0.004 (3)
C43	0.042 (4)	0.038 (4)	0.021 (4)	0.000 (3)	0.008 (3)	0.005 (3)
C44	0.039 (4)	0.036 (4)	0.039 (4)	0.005 (3)	0.003 (3)	0.002 (3)
C45	0.036 (4)	0.046 (4)	0.029 (4)	0.005 (3)	0.000 (3)	−0.007 (3)
C46	0.036 (4)	0.045 (4)	0.023 (4)	0.002 (3)	0.006 (3)	0.000 (3)
C47	0.032 (4)	0.036 (3)	0.025 (4)	0.008 (3)	0.001 (3)	0.001 (3)
C48	0.055 (5)	0.060 (4)	0.035 (4)	0.018 (4)	0.010 (4)	−0.003 (3)
N1S	0.117 (8)	0.105 (6)	0.075 (6)	0.003 (6)	−0.008 (6)	−0.030 (5)
C1S	0.115 (9)	0.053 (5)	0.062 (7)	−0.009 (6)	0.015 (7)	−0.017 (4)
C2S	0.090 (8)	0.065 (6)	0.112 (9)	0.011 (5)	0.025 (7)	−0.011 (5)

### Geometric parameters (Å, °)

**Table:**

Sm1—O2	2.302 (4)	C17—C18	1.382 (9)
Sm1—O3	2.321 (4)	C17—C20	1.507 (8)
Sm1—O1	2.346 (3)	C18—C19	1.384 (8)
Sm1—O4	2.432 (3)	C18—H18A	0.9500
Sm1—N2	2.556 (4)	C19—H19A	0.9500
Sm1—N3	2.605 (5)	C20—H20A	0.9800
Sm1—N4	2.671 (5)	C20—H20B	0.9800
Sm1—N1	2.753 (5)	C20—H20C	0.9800
O1—C13	1.301 (6)	C21—C22	1.440 (8)
O2—C27	1.293 (6)	C21—H21A	0.9500
O3—C41	1.326 (7)	C22—C23	1.395 (7)
O4—H4OA	0.9306	C22—C27	1.430 (8)
O4—H4OB	0.8259	C23—C24	1.376 (8)
N1—C5	1.469 (7)	C23—H23A	0.9500
N1—C3	1.476 (7)	C24—C25	1.402 (8)
N1—C1	1.488 (7)	C25—C26	1.373 (8)
N2—C21	1.277 (7)	C25—H25A	0.9500
N2—C2	1.454 (7)	C26—C27	1.406 (8)
N3—C7	1.288 (7)	C26—H26A	0.9500
N3—C4	1.473 (7)	C28—C29	1.379 (8)
N4—C35	1.283 (7)	C28—C33	1.384 (8)
N4—C6	1.495 (7)	C29—C30	1.379 (9)
N5—N6	1.261 (6)	C29—H29A	0.9500
N5—C10	1.416 (7)	C30—C31	1.380 (9)
N6—C14	1.429 (7)	C30—H30A	0.9500
N7—N8	1.255 (7)	C31—C32	1.386 (8)
N7—C24	1.426 (7)	C31—C34	1.514 (9)
N8—C28	1.435 (7)	C32—C33	1.380 (8)
N9—N10	1.270 (6)	C32—H32A	0.9500
N9—C38	1.413 (7)	C33—H33A	0.9500
N10—C42	1.438 (7)	C34—H34A	0.9800
C1—C2	1.521 (8)	C34—H34B	0.9800
C1—H1A	0.9900	C34—H34C	0.9800
C1—H1B	0.9900	C35—C36	1.463 (8)
C2—H2A	0.9900	C35—H35A	0.9500
C2—H2B	0.9900	C36—C41	1.381 (8)
C3—C4	1.498 (8)	C36—C37	1.401 (8)
C3—H3A	0.9900	C37—C38	1.377 (8)
C3—H3B	0.9900	C37—H37A	0.9500
C4—H4A	0.9900	C38—C39	1.417 (8)
C4—H4B	0.9900	C39—C40	1.362 (8)
C5—C6	1.509 (8)	C39—H39A	0.9500
C5—H5A	0.9900	C40—C41	1.419 (8)
C5—H5B	0.9900	C40—H40A	0.9500
C6—H6A	0.9900	C42—C47	1.375 (8)
C6—H6B	0.9900	C42—C43	1.393 (7)
C7—C8	1.443 (8)	C43—C44	1.382 (8)
C7—H7A	0.9500	C43—H43A	0.9500
C8—C9	1.397 (8)	C44—C45	1.387 (8)
C8—C13	1.418 (8)	C44—H44A	0.9500
C9—C10	1.386 (8)	C45—C46	1.388 (8)
C9—H9A	0.9500	C45—C48	1.517 (8)
C10—C11	1.400 (8)	C46—C47	1.376 (8)
C11—C12	1.360 (8)	C46—H46A	0.9500
C11—H11A	0.9500	C47—H47A	0.9500
C12—C13	1.433 (8)	C48—H48A	0.9800
C12—H12A	0.9500	C48—H48B	0.9800
C14—C15	1.378 (8)	C48—H48C	0.9800
C14—C19	1.387 (8)	N1S—C1S	1.132 (11)
C15—C16	1.393 (8)	C1S—C2S	1.456 (12)
C15—H15A	0.9500	C2S—H2S1	0.9800
C16—C17	1.391 (9)	C2S—H2S2	0.9800
C16—H16A	0.9500	C2S—H2S3	0.9800
			
O2—Sm1—O3	85.77 (14)	C16—C15—H15A	120.0
O2—Sm1—O1	76.50 (13)	C17—C16—C15	121.0 (6)
O3—Sm1—O1	82.33 (13)	C17—C16—H16A	119.5
O2—Sm1—O4	150.50 (12)	C15—C16—H16A	119.5
O3—Sm1—O4	83.01 (13)	C18—C17—C16	118.1 (6)
O1—Sm1—O4	75.01 (12)	C18—C17—C20	120.1 (6)
O2—Sm1—N2	69.91 (14)	C16—C17—C20	121.8 (6)
O3—Sm1—N2	123.51 (14)	C17—C18—C19	121.1 (6)
O1—Sm1—N2	134.52 (14)	C17—C18—H18A	119.4
O4—Sm1—N2	138.06 (13)	C19—C18—H18A	119.4
O2—Sm1—N3	94.97 (14)	C18—C19—C14	120.4 (6)
O3—Sm1—N3	150.32 (13)	C18—C19—H19A	119.8
O1—Sm1—N3	69.11 (13)	C14—C19—H19A	119.8
O4—Sm1—N3	81.97 (13)	C17—C20—H20A	109.5
N2—Sm1—N3	83.96 (14)	C17—C20—H20B	109.5
O2—Sm1—N4	125.88 (14)	H20A—C20—H20B	109.5
O3—Sm1—N4	68.93 (14)	C17—C20—H20C	109.5
O1—Sm1—N4	140.16 (13)	H20A—C20—H20C	109.5
O4—Sm1—N4	74.62 (12)	H20B—C20—H20C	109.5
N2—Sm1—N4	85.17 (14)	N2—C21—C22	126.7 (5)
N3—Sm1—N4	130.23 (15)	N2—C21—H21A	116.7
O2—Sm1—N1	131.48 (13)	C22—C21—H21A	116.7
O3—Sm1—N1	132.17 (13)	C23—C22—C27	119.9 (5)
O1—Sm1—N1	128.85 (13)	C23—C22—C21	116.3 (5)
O4—Sm1—N1	74.26 (12)	C27—C22—C21	123.7 (5)
N2—Sm1—N1	63.88 (14)	C24—C23—C22	122.1 (6)
N3—Sm1—N1	66.91 (14)	C24—C23—H23A	118.9
N4—Sm1—N1	64.71 (14)	C22—C23—H23A	118.9
C13—O1—Sm1	136.2 (3)	C23—C24—C25	118.2 (6)
C27—O2—Sm1	136.1 (4)	C23—C24—N7	124.9 (6)
C41—O3—Sm1	145.2 (3)	C25—C24—N7	116.7 (6)
Sm1—O4—H4OA	113.0	C26—C25—C24	120.7 (6)
Sm1—O4—H4OB	111.3	C26—C25—H25A	119.6
H4OA—O4—H4OB	105.1	C24—C25—H25A	119.6
C5—N1—C3	110.3 (4)	C25—C26—C27	122.3 (6)
C5—N1—C1	110.8 (4)	C25—C26—H26A	118.9
C3—N1—C1	108.8 (4)	C27—C26—H26A	118.9
C5—N1—Sm1	106.7 (3)	O2—C27—C26	123.0 (5)
C3—N1—Sm1	106.1 (3)	O2—C27—C22	120.5 (5)
C1—N1—Sm1	114.0 (3)	C26—C27—C22	116.6 (5)
C21—N2—C2	115.5 (5)	C29—C28—C33	119.2 (6)
C21—N2—Sm1	127.8 (4)	C29—C28—N8	116.2 (6)
C2—N2—Sm1	115.9 (3)	C33—C28—N8	124.5 (6)
C7—N3—C4	115.9 (5)	C28—C29—C30	120.5 (7)
C7—N3—Sm1	129.6 (4)	C28—C29—H29A	119.8
C4—N3—Sm1	114.5 (3)	C30—C29—H29A	119.8
C35—N4—C6	113.1 (5)	C29—C30—C31	121.1 (7)
C35—N4—Sm1	129.3 (4)	C29—C30—H30A	119.5
C6—N4—Sm1	117.4 (3)	C31—C30—H30A	119.5
N6—N5—C10	112.9 (5)	C30—C31—C32	118.0 (6)
N5—N6—C14	115.7 (5)	C30—C31—C34	121.8 (6)
N8—N7—C24	112.8 (5)	C32—C31—C34	120.2 (6)
N7—N8—C28	115.5 (5)	C33—C32—C31	121.5 (6)
N10—N9—C38	115.6 (5)	C33—C32—H32A	119.3
N9—N10—C42	112.1 (5)	C31—C32—H32A	119.3
N1—C1—C2	112.7 (5)	C32—C33—C28	119.7 (6)
N1—C1—H1A	109.1	C32—C33—H33A	120.1
C2—C1—H1A	109.1	C28—C33—H33A	120.1
N1—C1—H1B	109.1	C31—C34—H34A	109.5
C2—C1—H1B	109.1	C31—C34—H34B	109.5
H1A—C1—H1B	107.8	H34A—C34—H34B	109.5
N2—C2—C1	109.2 (5)	C31—C34—H34C	109.5
N2—C2—H2A	109.8	H34A—C34—H34C	109.5
C1—C2—H2A	109.8	H34B—C34—H34C	109.5
N2—C2—H2B	109.8	N4—C35—C36	127.6 (5)
C1—C2—H2B	109.8	N4—C35—H35A	116.2
H2A—C2—H2B	108.3	C36—C35—H35A	116.2
N1—C3—C4	112.3 (5)	C41—C36—C37	118.6 (6)
N1—C3—H3A	109.1	C41—C36—C35	124.3 (5)
C4—C3—H3A	109.1	C37—C36—C35	117.1 (6)
N1—C3—H3B	109.1	C38—C37—C36	122.1 (6)
C4—C3—H3B	109.1	C38—C37—H37A	118.9
H3A—C3—H3B	107.9	C36—C37—H37A	118.9
N3—C4—C3	110.4 (5)	C37—C38—N9	116.0 (5)
N3—C4—H4A	109.6	C37—C38—C39	118.4 (5)
C3—C4—H4A	109.6	N9—C38—C39	125.5 (5)
N3—C4—H4B	109.6	C40—C39—C38	120.4 (6)
C3—C4—H4B	109.6	C40—C39—H39A	119.8
H4A—C4—H4B	108.1	C38—C39—H39A	119.8
N1—C5—C6	112.3 (5)	C39—C40—C41	120.3 (5)
N1—C5—H5A	109.1	C39—C40—H40A	119.9
C6—C5—H5A	109.1	C41—C40—H40A	119.9
N1—C5—H5B	109.1	O3—C41—C36	121.7 (5)
C6—C5—H5B	109.1	O3—C41—C40	118.3 (5)
H5A—C5—H5B	107.9	C36—C41—C40	119.9 (6)
N4—C6—C5	110.2 (4)	C47—C42—C43	119.7 (6)
N4—C6—H6A	109.6	C47—C42—N10	116.8 (5)
C5—C6—H6A	109.6	C43—C42—N10	123.4 (5)
N4—C6—H6B	109.6	C44—C43—C42	119.1 (6)
C5—C6—H6B	109.6	C44—C43—H43A	120.5
H6A—C6—H6B	108.1	C42—C43—H43A	120.5
N3—C7—C8	127.6 (6)	C43—C44—C45	121.9 (6)
N3—C7—H7A	116.2	C43—C44—H44A	119.1
C8—C7—H7A	116.2	C45—C44—H44A	119.1
C9—C8—C13	119.7 (5)	C44—C45—C46	117.7 (6)
C9—C8—C7	118.1 (6)	C44—C45—C48	120.4 (6)
C13—C8—C7	122.2 (5)	C46—C45—C48	121.9 (6)
C10—C9—C8	122.3 (6)	C47—C46—C45	121.2 (6)
C10—C9—H9A	118.9	C47—C46—H46A	119.4
C8—C9—H9A	118.9	C45—C46—H46A	119.4
C9—C10—C11	118.1 (5)	C42—C47—C46	120.4 (5)
C9—C10—N5	117.3 (6)	C42—C47—H47A	119.8
C11—C10—N5	124.7 (6)	C46—C47—H47A	119.8
C12—C11—C10	121.2 (6)	C45—C48—H48A	109.5
C12—C11—H11A	119.4	C45—C48—H48B	109.5
C10—C11—H11A	119.4	H48A—C48—H48B	109.5
C11—C12—C13	121.8 (6)	C45—C48—H48C	109.5
C11—C12—H12A	119.1	H48A—C48—H48C	109.5
C13—C12—H12A	119.1	H48B—C48—H48C	109.5
O1—C13—C8	122.3 (5)	N1S—C1S—C2S	177.8 (12)
O1—C13—C12	120.9 (5)	C1S—C2S—H2S1	109.5
C8—C13—C12	116.8 (5)	C1S—C2S—H2S2	109.5
C15—C14—C19	119.2 (6)	H2S1—C2S—H2S2	109.5
C15—C14—N6	126.2 (6)	C1S—C2S—H2S3	109.5
C19—C14—N6	114.6 (5)	H2S1—C2S—H2S3	109.5
C14—C15—C16	120.1 (6)	H2S2—C2S—H2S3	109.5
C14—C15—H15A	120.0		

### Hydrogen-bond geometry (Å, °)

**Table:**

*D*—H···*A*	*D*—H	H···*A*	*D*···*A*	*D*—H···*A*
O4—H4OA···O1^i^	0.93	1.76	2.624 (5)	153
O4—H4OB···O3^i^	0.83	2.00	2.761 (5)	153

Symmetry codes: (i) −*x*+1, −*y*+1, −*z*+1.
